# Assessing trans fat levels in street foods of Tbilisi: a public health concern (2021)

**DOI:** 10.1186/s40795-025-01189-w

**Published:** 2025-11-12

**Authors:** Natia Kakutia, William Michael Caudle, Ziad N. Kazzi, Lela Sturua, Shalva Davit Zarnadze, Ekaterine Uberi, Nana Mebonia

**Affiliations:** 1 Department of Epidemiology and Biostatistics, Tbilisi State Medical University, Tbilisi, Georgia; 2 Gangarosa Department of Environmental Health, Rollins School of Public Health, Emory University, Atlanta, GA USA; 3 Department of Emergency Medicine, Emory University School of Medicine, Atlanta, GA USA; 4 Noncommunicable Diseases Department, National Center for Disease Control and Public Health, Tbilisi, Georgia; 5 Department of Nutrition, Aging Medicine, Environmental and Occupational Health, Tbilisi State Medical University, Tbilisi, Georgia; 6 Department of Therapeutics, Tbilisi State Medical University Pediatric Clinic, Tbilisi, Georgia

**Keywords:** Trans fats (TFA), Total fat, Street food

## Abstract

**Background:**

Industrially produced trans fats (TFA) are associated with significant public health risks, contributing to non-communicable diseases. The World Health Organization (WHO) recommends limiting TFA intake to less than 1% of total energy intake. However, street foods often contain high levels of TFA. Street food is widely consumed in Tbilisi, Georgia, but data on its TFA content is limited. This study assesses TFA levels in various street foods, providing detailed insights.

**Methods:**

Data were obtained from the 2021 Feed Cities Survey in Tbilisi. A random sample of 80 street food items was collected from 120 different vendors, with 20 food types, each sampled four times. TFA and total fat content were analyzed using gas chromatography. Descriptive statistics were calculated for both trans fat and total fat content per 100 g and per portion for each street food item. ANOVA, Pearson correlation and regression analyses were used to estimate differences between food categories and explore predictors of TFA levels.

**Results:**

The mean TFA content was 0.13 g per 100 g of food and 0.31 g per typical serving size provided by the street vendor. Khachapuri (Georgian traditional cheese bread) varieties had the highest TFA levels per portion, significantly differed from other food categories (*p* < 0.001). Total fat content per portion and portion size were positively associated with TFA levels (*r* = 0.66, *p* < 0.01 and *r* = 0.38, *p* < 0.01, respectively). Regression analysis showed total fat per portion as a significant predictor of TFA (*p* < 0.001), explaining 43% of the variance in TFA levels.

**Conclusions:**

Although Georgia’s regulations set a limit of 2 g of TFA per 100 g of fat, our findings show that some traditional street foods, particularly Khachapuri, may still contribute significantly to TFA intake. While TFA levels were below the regulatory limit, larger portion sizes and frequent consumption could exceed the WHO’s daily recommended intake. These results highlight the need for stricter enforcement, continuous monitoring and public health strategies to reduce trans fat intake, total fat content, and portion sizes.

## Background

According to the World Health Organization (WHO), trans fats (TFA) are unsaturated fats mainly produced industrially by partially hydrogenating vegetable oils. Small amounts of these fats can also be found in dairy and meat products. The WHO recommends that TFA intake should be less than 1% of total energy consumption and that the daily limit should be less than 2.2 grams. The WHO also states that “Industrially produced trans fats are not part of a healthy diet and should be avoided” [1]. Despite this, industrial trans fats remain common in many foods, with some processed items containing up to 60% of their total fat as trans fats [2].

Every year, industrial trans fats cause over 278,000 deaths worldwide [3]. They increase the risk of heart disease, diabetes, Alzheimer’s and other health issues [4–7]. Despite these risks, trans fats are still widely used in food production because they provide benefits like longer shelf life, better taste and improved texture. Many street food vendors use trans fats for deep-frying due to their stability and the ability to reuse them [8–10]. Currently, approximately 2.5 billion people globally consume street food daily [11].

Street food refers to “ready-to-eat foods and drinks sold by vendors in public places” and is an important part of urban diets, especially in developing countries [12]. Its popularity is growing due to changing eating habits and busy lifestyles that leave less time for home cooking [13]. While street food is affordable and convenient, it often contains high levels of trans fats. Many countries have regulations to limit trans fats in food production, but street food markets are often not regulated [11].The Retail in Georgia, 2019 report shows that fast-food restaurants are increasing in Georgia [14]. Street food has become a significant part of many Georgians’ diets, including traditional pastries filled with cheese, meat and beans - often made with partially hydrogenated margarine or vegetable fats – and Western-style fast foods like pizza and hamburgers. These fats are preferred for their lower cost and longer shelf life. Moreover, fried items such as meat- or potato-filled pastries are typically cooked in oils that are repeatedly reheated — a practice known to elevate trans fat levels.

Georgia’s laws limit trans fats to 2 g per 100 g of total fat (Government Decree N262, 2016; N353, 2017). However, the TFA content of these foods is not well documented. Since non-communicable diseases (NCDs) cause 93% of deaths in Georgia [15], it’s important to understand the risks of high trans fat intake for public health.

This study aims to measure the trans fat levels in various street foods in Tbilisi, providing detailed information on the TFA content of these widely consumed foods.

## Methodology

### Study design and data source

We used data from the cross-sectional FeedCities survey conducted in Tbilisi, Georgia during November and December 2021. This survey evaluated the nutritional composition of commonly consumed street foods, with a specific focus on chemically identifying and quantifying industrially produced trans-fatty acids and total fat content in ready-to-eat items. To collect a representative sample of street foods across the city, a comprehensive site selection strategy was implemented. All eligible street food vending sites, totally 371 in Tbilisi were identified and mapped. From them a sample of 120 was selected through simple random sampling. This method allowed for the inclusion of traditional and modern street foods.

Based on the popularity and variety of street foods commonly consumed by the local population, we chose 20 popular street food items for analysis. This selection was based on informal indicators including frequency of item availability and vendor-reported sales volume. To capture differences in preparation, each item was sampled from four randomly selected vendors out of 120 identified. Samples were collected in typical serving sizes, defined as single eating portions usually consumed by one person at one time. Following collection, any packaging materials were removed to ensure that the measured portion reflected only the edible component. Each sample was then weighed using a calibrated digital scale. These weights were used to calculate trans fat and total fat content per portion. Values expressed per 100 g refer exclusively to the single portion of the food, as determined after preparation and removal of non-edible components. After weighing, samples were homogenized, aliquoted, and stored at − 18 °C until analysis. Throughout the transportation process, all samples were maintained under controlled cold chain conditions to preserve their integrity. Chemical lab analysis of total fat and trans-fatty acid content was performed using gas chromatography, following internationally recognized ISO standards [16].

### Ethical considerations and consent to participate

This study involved the analysis of food samples collected from street food vending sites and did not involve direct human participation. As such, no individual human consent forms were required. The study protocol was reviewed and approved by the Ethics Committee of the National Center for Disease Control and Public Health (NCDC). All procedures were conducted in accordance with relevant ethical guidelines and institutional regulations.

### Study variables

The outcome variable of the study was the amount of trans fatty acids (TFA) per serving (g/portion). In addition, total fat levels were assessed to provide a broader understanding of the fat profile in the street foods. The independent (exposure) variables included 20 different food items, which were grouped into six categories based on their predominant ingredients and preparation methods. These categories were developed in the absence of a standardized national food composition database for street foods in Georgia and were therefore informed by culinary characteristics rather than formal classification systems. In this study, street foods were categorized based on their culinary origins. Traditional street foods included khachapuri varieties, lobiani, sweet and savory pastries, cakes, cookies, and bread products, all of which are commonly prepared and consumed within Georgian cuisine. By contrast, non-traditional fast foods referred to globally popular, Western-style items that are not native to Georgian culinary culture but are widely available in urban vending sites. These included pizza, hamburgers, hotdogs, and shawarma.These categories included: (a) four variations of Georgian khachapuri, which is a traditional Georgian dish of cheese-filled bread: Megruli khachapuri, Imeruli khachapuri, Guruli khachapuri, and layered khachapuri; (b) two types of Lobiani (Georgian bread filled with beans): traditional lobiani and lobiani with ham; (c) five types of sweet pastries: baklava, cream cake, loose cake/muffin, sweet puff pastry, and bun; (d) four types of savory baked goods: kubdari (traditional meat-filled bread), savoury meat pie, savory potato pie and chebureki (meat-filled fried pastry); (e) four types of non-traditional fast foods: hamburgers, hotdogs, shawarma, and pizza; (f) Georgian traditional bread (i.e., Lavashi).

To provide context for the fat content findings, it is known from local culinary practices that margarine or vegetable oils are commonly used in the preparation of pastries such as khachapuri, lobiani, and sweet pastries, contributing to higher trans fat levels. Similarly, deep-fried items like chebureki, savory meat pies, and potato pies are typically cooked in reused vegetable oils, which may elevate trans fat content. For non-traditional fast foods, industrially processed ingredients - including partially hydrogenated oils, processed meats, and commercially prepared sauces - are commonly used, which can contribute to their overall fat and trans fat content. However, detailed information on specific recipes or preparation methods for the sampled foods was not collected during this study, as the focus was on chemical analysis of fat content rather than food preparation processes.

### Data analysis

Descriptive statistics, including the mean and standard deviation, were calculated for both trans fat and total fat content per 100 g and per portion for each street food item. For each of the 20 food types, four individual samples were averaged to obtain a single value per food item. These item-level means were then used to compute category-level statistics and served as the analytical units in ANOVA and post-hoc tests, ensuring independent observations and valid comparisons between food categories.

To assess whether there were significant differences in trans fat and total fat content across food categories, an Analysis of Variance (ANOVA) was performed. The F-value was used to determine the overall significance of differences between categories, and a p-value of less than 0.05 was considered statistically significant. In cases where ANOVA indicated significant differences (*p* < 0.05), Tukey’s Honest Significant Difference (HSD) post-hoc test was applied to identify specific food categories that differed significantly in terms of their trans fat or total fat content.

Pearson correlation analyses were performed to examine the relationships between: (1) trans fat per portion and total fat per portion, and (2) trans fat per portion and portion size. To further explore how total fat content and portion size predict trans fat levels, a multiple regression analysis was conducted. In this model, trans fat per portion was the dependent variable, while total fat per portion and portion size were the independent variables. The overall significance of the regression model was evaluated using the F-statistic, with a p-value of less than 0.05 indicating that at least one of the independent variables significantly predicted trans fat levels. The regression coefficients (B) represented the change in trans fat per portion for a one-unit increase in the predictor variables. Standardized coefficients (Beta) were also calculated to reflect the change in the dependent variable in standard deviation units for each standard deviation increase in the predictors. The “Bread Products” category was excluded from the ANOVA, correlation, and regression analyses due to the absence of detectable trans fats in these items. Data analysis was conducted using SPSS Version 24.0 and Excel.

## Results

A total of 80 street food samples, representing 20 different food types, were analyzed, with each type sampled four times. The portion sizes varied widely, with a mean of 238.6 g (range: 26.7–700 g). The mean trans fat content was 0.13 g per 100 g and 0.31 g per portion, while the mean total fat content was 11.13 g per 100 g and 24.2 g per portion.

Khachapuri products had the highest trans fat content per 100 g, while bread products, like Lavashi, contained insignificant amounts. Similarly, Khachapuri varieties had the highest mean trans fat content per portion, whereas bread products showed no detectable trans fats. Sweet pastries had the highest total fat content per 100 g, while Khachapuri varieties had the highest total fat content per portion. Bread products like Lavashi showed the lowest total fat content, both per 100 g and per portion (Table 1).

**Table 1:** Mean ± SD of total fat and trans fat content by food category (*n* = 80)

Statistics	Overall (Mean (SD))	Khachapuri Varieties *n* = 16	Lobiani Varieties *n* = 8	Sweet Pastries *n* = 20	Savory Foods *n* = 16	Non-Traditional Fast Foods *n* = 16	Bread Products *n* = 4
Portion Size (g)	238.6 (114.8)	290.8 (62.6)	274.9 (55.7)	127.9 (50.1)	247.9 (66.8)	294.6 (181.5)	250.0 (0.0)
TFA per 100 g (g)	0.13 (0.11)	0.25 (0.14)	0.07 (0.05)	0.12 (0.09)	0.11 (0.09)	0.12 (0.09)	0.0 (0.0)
TFA per portion (g)	0.31 (0.31)	0.7 (0.4)	0.2 (0.1)	0.2 (0.1)	0.3 (0.2)	0.3 (0.2)	0.0 (0.0)
Total Fat per 100 g (g)	11.1 (6.35)	12.82 (5.41)	10.3 (5.74)	13.9 (7.03)	11.3 (6.60)	8.9 (3.61)	0.50 (0.34)
Total Fat per portion (g)	24.2 (14.9)	35.1 (10.8)	26.4 (11.6)	17.1 (11.4)	26.5 (15.4)	24.4 (15.6)	1.3 (0.8)

Food categories are based on culinary tradition and common classification in Georgia. “Khachapuri Varieties,” “Lobiani Varieties,” “Sweet”, “Savory Pastries” and “Bread” represent traditional Georgian street foods, while “Non-Traditional Fast Foods” refer to items not rooted in local cuisine but widely consumed and globally popular, such as pizza, hamburgers, hotdogs and shawarma. All values represent mean ± SD calculated across individual food items per category, with each item based on four analyzed samples

The ANOVA test revealed significant differences in trans fat content per portion across food categories, with an overall F-value of 13.5 (*p* < 0.001). Similarly, significant differences were observed in total fat content per portion (F-value = 4.2, *p* = 0.004) (Table 2).

**Table 2:** ANOVA results for trans fat and total fat content across all food categories

Dependent Variable	F-value	*p*-value
Trans fat per portion	13.5	*p* < 0.01
Total fat per portion	4.2	*p* < 0.01

Tukey’s HSD post-hoc test identified that Khachapuri varieties had significantly higher trans fat content compared to Lobiani, Sweet Pastries, Savory Foods and Non-Traditional Fast Foods. The largest difference in trans fat content was between Khachapuri and Sweet Pastries, with a mean difference of 0.6 g per portion (*p* < 0.001). Significant differences were also observed in total fat content per portion between Khachapuri and Sweet Pastries (mean difference = 17.9 g, *p* < 0.01), with Khachapuri containing higher total fat. No significant differences were found in other food categories (see Table 3).

**Table 3:** Post-Hoc results for trans fat and total fat across food categories

Dependent Variable	Comparision	Mean Difference	Std. Error	*p*-value (Tukey HSD)	95% Confidence Interval
Trans fat per portion	Khachapuri vs. Lobiani	0.5	0.1	*p* < 0.01	0.25–0.84
	Khachapuri vs. Sweet Pastries	0.6	0.1	*p* < 0.01	0.32–0.78
	Khachapuri vs. Savory Pastries	0.4	0.1	*p* < 0.01	0.19–0.68
	Khachapuri vs. Fast Food	0.4	0.1	*p* < 0.01	0.14–0.64
Total fat per portion	Khachapuri vs. Sweet Pastries	17.9	4.4	0.01	5.6–30.3

Pearson correlation coefficients were calculated to explore the relationships between trans fat per portion, portion size and total fat per portion. Significant positive correlations were found between trans fat per portion and total fat per portion (*r* = 0.66, *p* < 0.01) and a moderate correlation between trans fat per portion and portion size (*r* = 0.38, *p* < 0.01) indicating that higher total fat and larger portion sizes are associated with increased trans fat levels (Table 4).

**Table 4:** Pearson correlation coefficients (Significant at *p* < 0.01 level)

	Trans Fat per Portion (g)	Portion Size (g)	Total Fat per Portion (g)
Trans Fat per Portion		0.4*	0.7*
Portion Size (g)	0.4*		0.5*
Total Fat per Portion (g)	0.7*	0.5*	

*Significance level: *p* < 0.01

Regression analysis further explored these relationships, revealing that total fat per portion is a significant predictor of trans fat per portion (*p* < 0.001), explaining 43% of the variance in trans fat levels. Total fat per portion was a significant positive predictor (B = 0.014, *p* < 0.001), indicating that for every 1-gram increase in total fat per portion, trans fat per portion increased by 0.014 g. Portion size was not a significant predictor (B = < 0.001, *p* = 0.59) (Table 5).

**Table 5:** Regression analysis summary predicting TFA per portion based on portion size and total fat

Model	*R*	*R*²	Adjusted *R*²	Std. Error of the Estimate	F	Sig. F
1	0.7	0.4	0.4	0.2	27.	0.000

Predictor	B	Std. Error	Beta	t	Sig.	
(Constant)	−0.06	0.07		−0.8	0.39	
Portion Size (g)	0.0	0.00	0.06	0.54	0.59	
Total Fat per Portion	0.014	0.002	0.63	6.09	0.00	

F: Overall significance of the regression model

Sig. F: Significance of the F-statistic, indicating the model’s overall fit

## Discussion

Street food categories contribute differently to the WHO’s daily trans fat limit of 2.2 g. The trans fat content in these foods ranges from 8% to 33% of this limit. One item from the “Khachapuri Varieties” category reached 45%. Given that it is common for people in Georgia to consume traditional foods like khachapuri, lobiani and kubdari multiple times a day, their trans fat intake can easily exceed the recommended daily limit, especially with larger portion sizes.

Our study found a link between larger portion sizes and higher trans fat intake. However, the analysis showed that portion size itself was not a significant predictor of trans fat levels, suggesting that other factors, such as the type and quality of fats used, play a more important role. Many food items contained enough trans fat per portion to account for a significant portion of the daily recommended limit. Frequent consumption of these foods could easily lead to exceeding the WHO’s guidelines, posing serious health risks. The use of trans fats in street foods is not unique to Tbilisi. It is a common issue in urban areas worldwide. For example, studies from Bosnia and Herzegovina, India and Central Asia have found high trans fat levels in street foods due to the widespread use of partially hydrogenated margarine and other fats [17–19]. In Moldova, industrial fats have also been linked to elevated trans fat levels [20].

Additionally, our study found that street foods with more total fat often have higher trans fat levels. Foods high in total fat usually contain ingredients like butter, oil, margarine, cheese, or other fatty components. Partially hydrogenated margarine and oils are especially common in pastries and fried foods, which are popular street food choices. Reusing frying oil also increases trans fat levels and worsens the problem [21].

Our analysis showed that total fat content correlates with trans fat levels, as expected since trans fat is a subset of total fat. Regression analysis indicated that total fat per portion explains 43% of the variance in trans fat content, but the remaining variance highlights the importance of fat type and quality. Ingredients such as margarine, reused oil, and certain dairy products contribute substantially to elevated trans fat levels. This emphasizes the need for public health interventions focused not only on reducing total fat but also on improving fat composition.

Focusing more closely traditional street foods in Tbilisi, like khachapuri, high total fat content may result from extensive use of butter, cheese, or oil. These ingredients contribute to both total fat and trans fat levels, especially when partially hydrogenated margarine or other fats are used. However, our data showed that some foods with high total fat content had relatively low trans fatty acid (TFA) levels. This suggests that while these foods contain a lot of fat, the type of fat used does not always increase trans fat levels. Food categories such as lobiani, cakes, cookies, sweet pastries, savory baked goods and non-traditional fast foods had lower trans fat levels than khachapuri varieties. However, they still exceeded recommended limits when considering daily intake. These differences may be due to variations in cooking methods and ingredient choices.

### Study limitations

This study focuses on street foods in Tbilisi, as it was our intention to provide insights specific to this region and the findings may not apply to other parts of Georgia. The small sample size for each food category limits the generalizability of our results. Additionally, variations in recipes and cooking methods could have influenced trans fat levels, which were not fully captured in this study. Another consideration is that trans fat is a subcategory of total fat; however, the proportion of trans fat varies substantially depending on the types of fats and oils used in food preparation. In the Georgian context, many street foods are made with partially hydrogenated margarine or fats known to contain high levels of industrially produced trans fats, while deep-fried items often involve reused oils. Therefore, trans fat content cannot be assumed as a fixed fraction of total fat, emphasizing the need for specific quantification of trans fats.

Despite these limitations, this study provides a comprehensive assessment of trans fat content in street foods specifically in Tbilisi. It provides important insights into urban eating habits and public health issues. By including different street food categories, we can analyze trans fat levels in foods that are commonly consumed, making the findings more relevant to local consumers.

## Conclusion

Despite Georgia’s regulations allowing a maximum of 2 g of TFA per 100 g of fat, our findings show that some traditional street foods, especially Khachapuri varieties, can still contribute a lot to total trans fat intake. Although the TFA levels were below 2 g per 100 g in our study, larger portion sizes and frequent consumption could lead to exceeding the WHO’s recommended TFA daily limit of 2.2 g.

While portion size wasn’t statistically linked to trans fat levels, other factors, like the type of fats used, have a bigger impact. This highlights the need for better enforcement and ongoing monitoring. Stricter regulations and promoting healthier cooking fats could help reduce trans fat intake. Additionally, these findings show the importance of public health strategies to reduce trans fats and improve the nutritional quality of street foods in Georgia. Public health interventions focused on lowering total fat content and portion sizes, along with regular monitoring, are key to improving dietary quality and reducing the health risks of trans fats.

## Data Availability

The data that support the findings of this study are available from the corresponding author upon reasonable request.

## Abbreviations

WHO: World Health Organization
TFA: Trans Fatty Acids
NCDs: Non-Communicable Diseases
NCDC: National Center for Disease Control and Public Health
ANOVA: Analysis of Variance
HSD: Honest Significant Difference
SPSS: Statistical Package for the Social Sciences
SD: Standard Deviation
